# Cannabis-Based Products in a Neurological Setting: A Clinical and Pharmacokinetic Survey

**DOI:** 10.3389/fneur.2022.784748

**Published:** 2022-03-24

**Authors:** Susan Mohamed, Giovanna Lopane, Loredana Sabattini, Cinzia Scandellari, Diletta Zardi, Vincenzo Donadio, Giovanni Rizzo, Alessandro Perrone, Alessandra Lugaresi, Manuela Contin

**Affiliations:** ^1^IRCCS Istituto delle Scienze Neurologiche di Bologna, Bologna, Italy; ^2^Department of Biomedical and Neuromotor Sciences, University of Bologna, Bologna, Italy

**Keywords:** cannabidiol, delta-9-tetrahydrocannabinol, cannabis oil, cannabis oromucosal spray, neurology, pharmacokinetics, medical cannabis

## Abstract

**Background and Aim:**

Limited data are available in clinical settings on the pharmacokinetics of delta-9-tetrahydrocannabinol (THC) and cannabidiol (CBD). We investigated the use of cannabis-based products in neurological practice, monitoring patients' steady-state cannabinoids (CBs) plasma concentrations matched with different preparations.

**Methods:**

This was a prospective, single-center, observational study. Patients underwent venous blood withdrawal before the CBs' morning dose and then 2.5 h post-dosing. Spasticity or pain were patient self-assessed by the Numeric Rating Scale (NRS) before the morning CB's administration and 2.5 h post-dosing.

**Results:**

Thirty-three patients were enrolled. Main indications for CBs were spasticity and chronic pain. Sixteen patients were treated with oromucosal spray formulation Sativex® and 17 with oil-based solutions. Both CBs trough plasma concentrations were ≤ limit of detection (0.1 ng/ml) in 45% of patients. Intrasubject CB's plasma levels significantly increased over baseline values in patients treated with Bediol® oil (*p* < 0.05) and Sativex® (*p* < 0.01). Post-dosing CB's bioavailability did not significantly differ between oral oil and oromucosal spray. NRS scores decreased (*p* < 0.01), matching the increase (*p* < 0.01) in CB's plasma concentrations.

**Conclusion:**

This is the first study investigating CB's plasma concentrations of oral and oromucosal preparations in real-world neurological practice. Findings of similar bioavailability for both CBD and THC after galenic oil compared with oromucosal spray dosing may be clinically relevant and deserve additional research in larger cohorts.

## Introduction

The cannabis plant contains several substances, such as more than one hundred cannabinoids (CBs) (1). There is great interest in the use of cannabis for the management of many diseases and symptoms (2) and the attention has been focused in particular on the two CBs, delta-9-tetrahydrocannabinol (THC) and cannabidiol (CBD) (3).

Two types of CB receptors have been identified, CB1 and CB2, which are parts of the human endocannabinoid system, involved in various functions, such as muscle spasticity, analgesic activity, anticonvulsant properties, vasodilatory and hypotensive action, and appetite (3).

THC is a partial agonist of CB1 and CB2 receptors. The main pharmacological effects of THC are psychoactivity, analgesia, muscle relaxation, anti-vomiting, and stimulation of appetite (3). Muscle relaxant effects are also recognized for hyper-reflexic bladder (4, 5).

Cannabidiol does not have a direct effect on the CB1 or CB2 receptors responsible for cannabis psychoactivity but it has been shown to have a negative allosteric activity on CB1 (6). From experimental models of epilepsies, different CBD mechanisms have emerged, such as antagonism of G protein-coupled receptor 55 (GPR55), desensitization of transient receptor potential of vanilloid type 1 (TRPV1) channels, and interactions with voltage-gated sodium and potassium channels (7). Other mechanisms associated with anti-inflammatory pathways include direct agonistic activity on serotonin 1A and adenosine A2A receptors (8). The main pharmacological effects of CBD are antiseizure, muscle relaxant, anxiolytic, and anti-inflammatory (1).

In the literature, several works have examined the efficacy and safety of CB's preparations in the treatment of a series of symptoms associated with neurological diseases, such as multiple sclerosis (MS), epilepsies, Huntington's disease, Parkinson's disease, cervical dystonia, and Tourette's syndrome. These applications were reviewed by an *ad hoc* guideline development subcommittee of the American Academy of Neurology (9). Chronic pain is another field of use of therapeutic cannabis, although few rigorous studies have evaluated its effectiveness (10).

Available data on CB's safety from clinical trials and real-world experience show that the most common adverse effects (AEs) associated with THC are dizziness, drowsiness, dry mouth, nausea/vomiting, impairment in cognitive function (perception disorders, euphoria, and confusion) and psychomotor skills, balance, and coordination problems (11). Studies on recreational cannabis have suggested a link between early, frequent use of high potency THC, and earlier onset of psychosis in subjects with a personal or family history of schizophrenia or psychotic disorders (12). CBD's common AEs include diarrhea, somnolence, pyrexia, decreased appetite, vomiting, and upper respiratory tract infection (11).

In Italy, two cannabis medicinal products are authorized: one based on THC and CBD, in the approximate 1:1 dose ratio (2.7 mg THC and 2.5 mg for CBD), in a spray for oral mucosa (Sativex®, GW Pharmaceuticals, UK), indicated to relieve symptoms in adult patients who suffer from moderate to severe spasticity due to MS. The other, marketed from the end of June 2021, is based on purified plant-based CBD, in an oil oral formulation (Epidiolex, GW Pharmaceuticals, UK), indicated as an adjunct treatment of seizures associated with two rare, severe forms of epilepsy with childhood onset, the Lennox-Gastaut and Dravet syndromes. Furthermore, there are several available cannabis galenical preparations (i.e., oil extracts, decoctions) characterized by different percentages of THC and CBD (Supplementary Table 1), which can be prescribed by physicians to users registered on the Italian Ministry of Health database (13). Eligible indications of medical cannabis include the management of the chronic pain associated with MS and spinal cord injury, the control of nausea and vomiting due to chemotherapy, radiotherapy, or HIV therapy, the handling of appetite loss in oncologic and HIV-positive patients. Medical cannabis is also indicated for its appetite stimulant effect in cachexia and anorexia, its hypotensive effect in glaucoma, and as antispasmodic in Tourette's syndrome (14).

Despite the substantial number of published studies, data on the pharmacokinetics of THC and CBD are limited (15, 16). In particular, the pharmacokinetics of CBs from oral galenical preparations has not been extensively studied in clinical settings (17). CB's posological protocols for physicians are missing (14), thus, currently the management of dosing is largely empirical, based on a balance between the desired therapeutic effects and the prevention of the adverse ones. Knowledge of CB's pharmacokinetics from different available formulations could help the prescribers in optimizing therapeutic regimens.

The purpose of this study was to investigate the use of cannabis-based products at the Institute of Neurological Sciences of Bologna (ISNB), such as indications, type of patients treated, formulations, dosages, evidence of efficacy, and AEs, and to monitor steady-state CB's plasma concentrations matched with different cannabis-based products.

## Materials and Methods

### Study Design and Patients

This is a prospective, single-center, observational study. The study protocol was approved by the local Ethics Committee (CE 19030) and written informed consent was obtained from patients. The sample size was based on patient enrolment and not pre-calculated. Participation in the protocol was proposed to patients referring to the ISNB who were in therapy with cannabis-based products. Inclusion criteria were as follows:

(1) aged 18 years or older;

(2) stable treatment with a cannabis-based product for at least 1 month; no change in dosage of CBs over the preceding 3 weeks;

(3) written informed consent.

### THC and CBD Plasma Specimen Collection and Quantitation

Venous blood samples (3 ml) were drawn from patients between 8 and 9 am, median 12 h apart from the last evening dose and then 2.5 h after ingestion of their usual morning dose, taken after breakfast (basically, milk, or milk and coffee, or coffee or tea with pastry).

Blood samples were transferred into heparinized tubes and immediately centrifuged at 1,500 × g for 10 min, at 4°C. Separated plasma samples were stored at −80°C until analysis, within 6 months from the collection (18). Plasma concentrations of THC and CBD were measured by ultra-high-pressure liquid chromatography-mass spectrometry (UHPLC-MS-MS) based on Dulaurent et al. (19). The method originally developed for THC was internally implemented and validated by the Laboratory of Clinical Neuropharmacology of ISNB also for CBD plasma analysis. Sample pretreatment was the same, more precisely, 50 mg of QuEChERS salts (magnesium sulfate/sodium chloride/sodium citrate dehydrate/sodium citrate sesquihydrate) and 200 μl of acetonitrile containing deuterated CBs (THC-D3 and CBD-D3) as internal standards were added to 100 μl of plasma. Then the mixture was shaken and centrifuged for 10 min at 12,000 × g. Finally, 10 μl of the upper layer was injected into the UHPLC-MS-MS system for the analysis. Validation was carried out according to the European Medicines Agency guidelines (20). Linearity was checked over the range of 0.5–20 ng/ml for both THC and CBD, recovery averaged 75% for both analytes. The lower limit of quantification (LLOQ) and limit of detection (LOD) were 0.5 and 0.1 ng/ml, respectively, for both CBs. Intra- and interassay imprecision and inaccuracy were ≤15%.

### CB's Treatment Efficacy and Tolerability Assessment

Efficacy and tolerability of chronic CB's treatment were assessed on the morning of the study by clinical examination and patients' direct interview adopting a standardized case report form.

On the same morning, patients were asked to self-assess their main disease symptoms (spasticity or pain) by the Numeric Rating Scale (NRS) (21, 22) before the first morning administration of CBs and 2.5 h post-dosing, concomitantly with blood specimen collection.

### Data and Statistical Analysis

The main study outcome was the assessment of THC and CBD plasma concentrations. CB's post-morning dose bioavailability was expressed as the ratio between plasma concentration (C) and weight-adjusted administered dose (D): (C/D) [(ng/ml)/(mg/kg)]. Trough CBs, plasma concentrations were subtracted to matched post-dosing values to correct for residual levels coming from previous evening dose intake. Statistical analysis was performed by non-parametric tests. The statistical significance of variable differences between patients treated with Bediol® vs. Sativex® was assessed by the Mann-Whitney Rank Sum test. Comparisons of intrasubject pre- and post-dosing CB's plasma concentrations were performed by the Wilcoxon Signed Rank Test. Correlations between variables were assessed by Spearman's product-moment coefficient. Significance was set at *p* < 0.05. Analyses were carried out using SigmaPlot 12.5 software (Systat Software, San Jose, CA, USA).

## Results

### Clinical and Therapeutic Characteristics of Patients

Between April 12, 2019 and August 28, 2020, we enrolled 33 patients (16 women, 17 men), aged 55 ± 13 years (mean ± SD) receiving CB products. Main indications for CB prescription were spasticity in MS and hereditary spastic paraplegia and chronic pain conditions, especially neuropathic pain. Clinical details of patients grouped according to different cannabis-based products are reported in Table 1. Sixteen patients were treated with Sativex® and 17 with cannabis-based oily solutions: Bediol® (*n* = 10), Bedrocan® (*n* = 4), and Bedrolite® (*n* = 3). The median number of administrations were six a day (25–75 percentiles, 3–8) for Sativex® and twice a day (2–3) for galenical preparations. All galenics were prepared following Romano and Hazekamp's indications (23).

**Table 1:** Patients' characteristics associated with cannabinoid-based preparations grouped by type of disease.

**Therapeutic indications**	**N**°**of patients**	Age (years)^*^	**Sex m/f**	Weight (kg)^*^	**CBs treatment duration (months)^#^**	**CBs product (n)**
Spasticity in Multiple sclerosis	22	55 ± 13 33–78	13/9	75 ± 16 51–101	12 4.5–24	Sativex® (16)
						Bedrolite ® (1) Bedrocan® (2) Bediol® (3)
Spasticity in hereditary spastic paraplegia	3	46 ± 12 32–60	1/2	61 ± 9 48–68	5 2.5–10.5	Bedrolite® (2)
						Bediol® (1)
Chronic pain in: - Neuropathic pain - Parkinson's disease - Fibromyalgia + neck pain + headache	8 4 3 1	55 ± 14 35–74	3/5	67 ± 15 44–88	6 3–18	Bediol® (6) Bedrocan® (2)

Data are expressed as mean ± SD (*) and range or median and 25–75 percentiles (#); f, females; m, males; CBs, cannabinoids.

Other cotherapies were taken by 26 patients: Selective Serotonin Reuptake Inhibitors (SSRIs) antidepressants (*n* = 10); immunosuppressive drugs (azathioprine, *n* = 7); antihypertensives (beta blocking agents and calcium antagonists, *n* = 7); drugs used for neuropathic pain (gabapentin, *n* = 2 and pregabalin, *n* = 1) and trigeminal neuralgia (carbamazepine, *n* = 2); mood stabilizers (sodium valproate, n = 1); antiparkinsonian drugs (*n* = 4); muscle relaxant drugs (*n* = 4); and non-selective blocker of several voltage-sensitive potassium channels (4-aminopyridine, *n* = 6).

### CB's Plasma Concentrations

**Figure 1:**
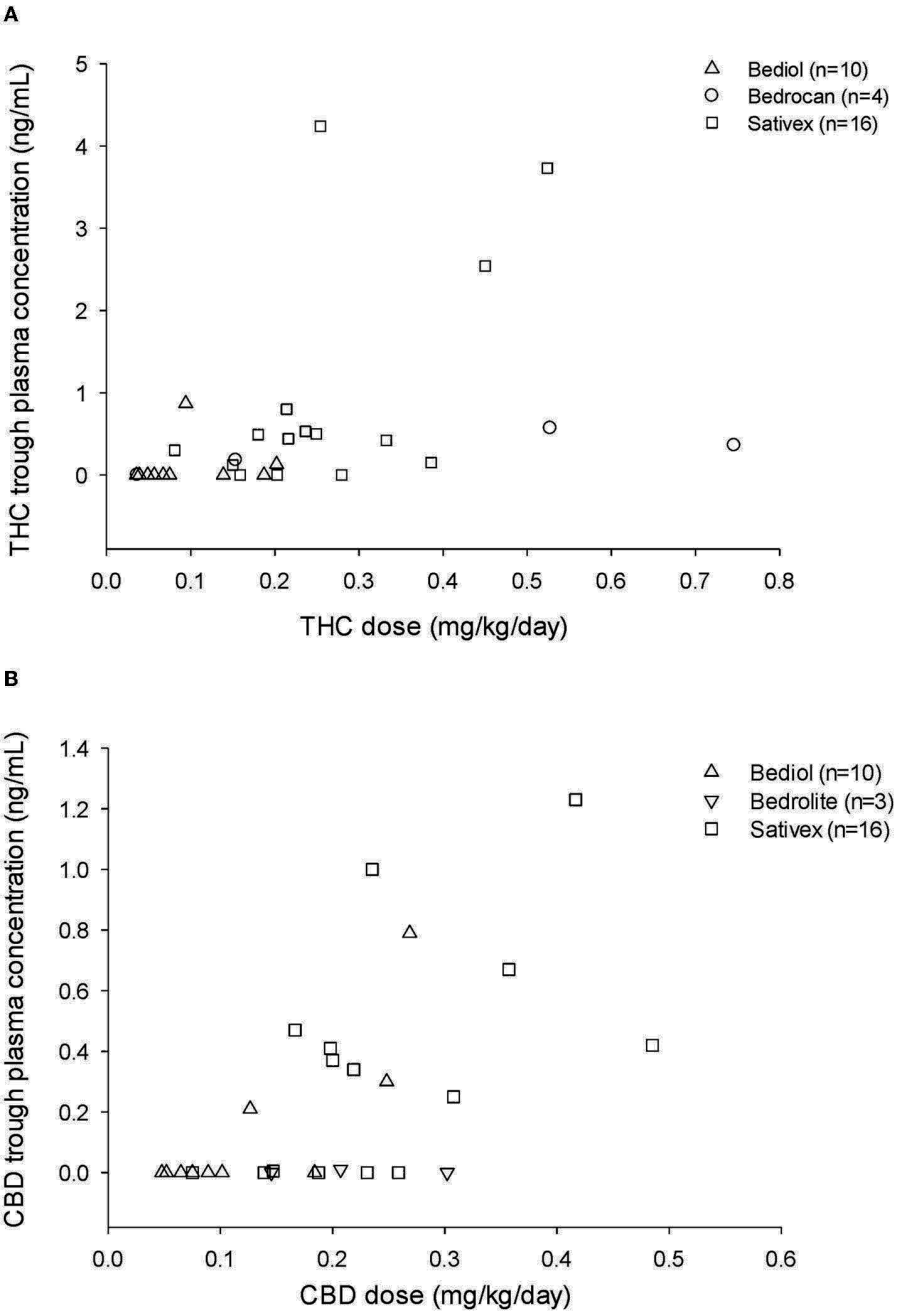
Tetrahydrocannabinol (THC) **(A)** and cannabidiol (CBD) **(B)** trough plasma concentrations and matched daily doses (mg/kg/day) by different types of cannabis-based products.

Cannabinoid's median daily doses and matched trough plasma concentrations are reported for different formulations in Table 2A. Both THC and CBD trough plasma concentrations were ≤ LOD in 8 out of 10 patients on Bediol®, 3 out of 16 on Sativex®, 1 out of 4 on Bedrocan®, and 3 out of 3 on Bedrolite®. A scatter plot of CBs trough plasma concentrations matched with daily doses per body weight for each cannabis-based product is shown in Figure 1.

**Table 2A:** Cannabinoids daily doses and matched trough plasma concentrations by different cannabis-based oral formulations.

	**Daily dose**		
	**(mg)**	**(mg/kg)**	**Trough plasma concentrations (ng/mL)**
THC in Bediol®	4.7	0.07	0
(*n* = 10)	(4–9)	(0.05–0.15)	(0–0.03)
THC in Sativex®	17.55	0.23	0.46
(*n* = 16)	(16.2–21.6)	(0.19–0.32)	(0.13–0.76)
THC in Bedrocan®	18.35	0.34	0.27
(*n* = 4)	(5.1–38.8)	(0.07–0.7)	(0.04–0.52)
CBD in Bediol®	6.3	0.09	0
(*n* = 10)	(5.35–12)	(0.06–0.2)	(0–0.23)
CBD in Sativex®	16.25	0.21	0.35
(*n* = 16)	(15–20)	(0.17–0.3)	(0–0.46)
CBD in Bedrolite®	14.5	0.21	0
(*n* = 3)	(9.6–14.5)	(0.14–0.3)	

**Figure 2:**
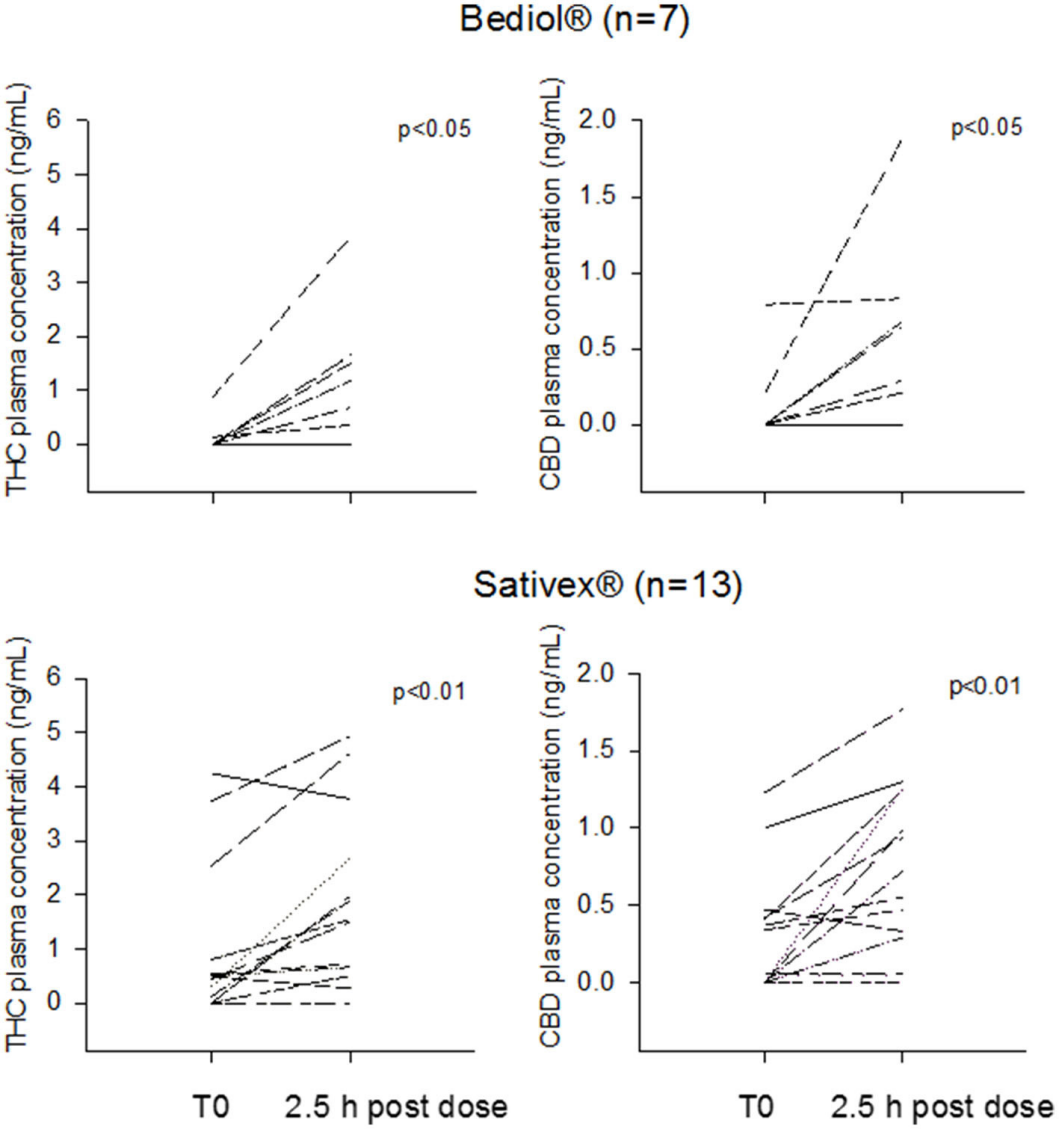
Intrasubject tetrahydrocannabinol (THC) and cannabidiol (CBD) morning trough and 2.5 h post-dosing plasma concentrations grouped by patients treated with Bediol® and Sativex®.

Post-morning dose plasma CB levels were available in 25 out of 33 patients (Table 2B). Intrasubject CB plasma levels 2.5 h post-dosing significantly increased over baseline values in patients treated with Bediol® (*n* = 7) and Sativex® (*n* = 13; Figure 2).

**Table 2B:** Cannabinoids morning doses, matched trough and post-dosing plasma concentrations in a subset of patients by different cannabis-based oral formulations.

	**Morning dose**		**Trough plasma concentration**	**Post morning dose plasma concentrations**	***p* <**
	**(mg)**	**(mg/kg)**	**(ng/mL)**	**(ng/mL)**	
THC in Bediol®	2.15	0.03	0	1.18	0.05
(*n* = 7)	(2.13–2.57)	(0.02–0.05)	(0–0.13)	(0.35–1.66)	
THC in Sativex®	5.4	0.07	0.49	1.55	0.01
(*n* = 13)	(4.72–8.1)	(0.05–0.1)	(0.06–1.67)	(0.58–3.22)	
THC in Bedrocan®	14.37	0.22	0.27	1.72	-
(*n* = 2)	(6.75–22)	(0.08–0.37)	(0.18–0.36)	(1.27–2.18)	
CBD in Bediol®	2.85	0.04	0	0.65	0.05
(*n* = 7)	(2.85–3.42)	(0.03–0.07)	(0–0.21)	(0.21–0.83)	
CBD in Sativex®	5.0	0.06	0.34	0.72	0.01
(*n* = 13)	(3.75–7.5)	(0.05–0.08)	(0–0.44)	(0.31–1.25)	
CBD in Bedrolite®	4.0	0.07	0	0	-
(*n* = 3)	(3.2–4.83)	(0.05–0.1)		(0–0.18)	

*Data are expressed as median (25–75 percentiles); THC, delta-9-tetrahydrocannabinol; CBD, cannabidiol; p, statistical significance of intrasubject comparisons between trough and post-morning dose plasma concentrations by the Wilcoxon Signed Rank Test*.

Post-dosing CB's bioavailability did not significantly differ between Bediol® and Sativex®, with median C/D values (25–75%), respectively, of 9.16 (0.60–20.1) vs. 4.32 (1.35–12.1) for CBD (*p* = 0.662) and 30.0 (4.37–67.9) vs. 7.51 (1.93–22.5) for THC (*p* = 0.189).

### CB's Treatment Efficacy and Tolerability Assessment

Spasticity or pain patients' self-assessment by NRS matched with morning CBs dosing was available in 23 patients. Clinical and therapeutic characteristics of these patients are reported in Supplementary Table 2. Overall NRS scores decreased from a median baseline value of 7 to 5 at 2.5 h post-dosing (*p* < 0.01), paralleling the significant increase (*p* < 0.01) in CB plasma concentrations (Supplementary Figure 1).

From clinical evaluation and patients' interview, the efficacy of chronic CB administration on spasticity and/or pain symptoms control was reported in all the enrolled patients but one with neuropathic pain treated with Bediol®. Fourteen patients (42%) reported one or more AEs regardless of the type of product: drowsiness (50%), behavioral changes (36%), such as agitation, hyperactivity, irritability, and constipation (14%). Burning in the oral mucosa (14%) was the only AE specific for Sativex®. These AEs were minor and considered tolerable by both patients and clinicians from the evaluation of the risk/benefit ratio of CB treatment. No subacute dosing AEs were reported by any patient.

## Discussion

The main considerations from this exploratory study on the use of CBs in a neurological tertiary clinical center are the following:

- Spasticity in MS was by far the most frequent therapeutic indication for CB prescription, followed by neuropathic chronic pain. We realized that despite neurologists' interest in the therapeutic applications of CB, their use is still limited in the clinical setting. The administrative burden for CB prescriptions, the lack of posological protocols, insufficient scientific evidence of their efficacy and tolerability, especially in the long term, complicate physicians' handling of these formulations (14).

Sativex® oromucosal spray specifically authorized for MS patients was the most frequently prescribed formulation. Among galenic oils, Bediol® was the most common. Both Sativex® and Bediol® are characterized by an approximate THC:CBD 1:1 content, a CB dose ratio suggested for pathological conditions, such as MS and pain (14). Bedrocan® and Bedrolite® oils, based almost exclusively on THC and CBD, respectively, were prescribed in a minority of patients. As previously pinpointed (14), physicians' choice of the kind of galenic formulation, THC:CBD ratio and doses proved largely empirical, based on an individualized “trial and error” approach, following the rule “start low, go slow, and stay low” (24).

- Baseline CB's plasma levels at a 12-h distance from the last dose intake were undetectable or near the LLOQ in the majority of our patients, especially those taking oral galenics. This is in keeping with the very low peak plasma concentrations of THC and CBD attained at currently proposed therapeutic doses and the twice a day dosing mostly adopted with oral oils. Reported CB's plasma half-lives vary largely among studies. Values ranging 1.94–3.72 h for THC and 5.28–6.39 h for CBD were reported after single-dose oromucosal intake (25). THC and CBD single doses in oil were associated with mean plasma half-lives ranging from 1.58 (26) to up to 28 h for THC (27) and 0.92 (26) to 3.3 h for CBD (27). These discrepancies may be ascribed to differences in LLOQ among analytical techniques, blood sampling times, and the use of appropriate weighting factor when fitting data using non-linear regression analysis (28). With chronic use, prolonged half-life values of CBs have been reported, possibly due to their gradual release from not defined deep compartments, such as adipose tissue (15).

Overall, post-morning dose of THC and CBD plasma concentrations significantly increased over baseline values. Blood drawings were taken 2.5 h post-dose, on the basis of our previous findings from oromucosal spray (29), and reported CBs t_max_ from oils ranging 1.3–2.5 h (16, 17, 27). In line with published data at comparable doses (26, 29, 30) CB's plasma concentrations proved low, up to maximum THC and CBD values of 3.86 and 1.88 ng/ml, respectively from oils and 4.94 and 1.77 ng/ml from oromucosal spray. Values lower than the suggested minimum effective CB's blood concentrations of 1 ng/ml (26) were measured in 43% of patients after Bediol® and 38% after Sativex® dosing for THC and in 86% of patients after Bediol® and 69% after Sativex® for CBD.

- Sativex® and Bediol® intake was associated with similar post-dosing CB bioavailability. The oromucosal route has been proposed to overcome the low and highly variable bioavailability of oral CB preparations, partly ascribable to high first pass metabolism (15). However, oromucosal doses may be partly ingested and absorbed in the esophagus and stomach and subjected to first-pass hepatic metabolism as well (29). Moreover, lesions of oral mucosa due to the spray alcohol content, a recognized AE of Sativex®, might affect CB disposition as well. In any case, these findings should be taken with caution due to the limited number of patients and the high intersubject variability in CB's plasma concentrations at a given dose, observed both with Sativex® and Bediol®. Many factors can contribute to this generally recognized variability, such as subject characteristics (i.e., age, body composition) (31, 32), drug peculiar pharmacokinetics (15, 24), pharmaceutical characteristics (i.e., poor CB's recovery in oils and variable stability over time) (33, 34).

- Despite the low CB plasma concentrations attained after dose intake, patients' self-reported NRS significantly improved over baseline ratings, paralleling the increase in CB plasma concentrations. These observations were mostly related to patients with MS (83%), on oromucosal spray in 68% of cases, and are in keeping with our previous pilot study on THC/CBD oromucosal plasma concentration – NRS effect relationship in MS (29).

## Conclusion

Albeit limited to a relatively small cohort, which was in any case comparable to the number of patients of previously published reports on this topic, the present study is the first to explore and compare THC and CBD plasma concentrations of oral and oromucosal CB preparations in real-world patients with neurological disorders. In particular, findings of similar bioavailability for both CBD and THC after galenic oil compared with oromucosal spray dosing may have clinical practical implications and deserve additional research in larger cohorts.

## Data Availability Statement

The raw data supporting the conclusions of this article will be made available in Zenodo.org, DOI “10.3389/fneur.2022.784748”, https://zenodo.org/record/6346371#.YjBeC-jMKUl.

## Ethics Statement

The studies involving human participants were reviewed and approved by the Comitato Etico di Area Vasta Emilia Centro della Regione Emilia-Romagna (CE-AVEC). The patients/participants provided their written informed consent to participate in this study.

## Author Contributions

SM, GL, LS, CS, VD, GR, AL, and MC contributed to the conception and design of study protocol. SM, GL, DZ, LS, and AP acquired the study data. SM organized the database and performed quantitation analyses of plasma cannabinoids. SM and MC performed the statistical analysis and wrote the first draft of the manuscript. All authors contributed to the interpretation of data and manuscript critical revision.

## Funding

This study was partly financed with contributions of 5 x 1000- Support for Neuroscience Health Research – 2016 of IRCCS Istituto delle Scienze Neurologiche di Bologna, Bologna, Italy.

## Conflict of Interest

AL has served as a Biogen, Merck Serono, Novartis, Roche, Sanofi/Genzyme Bristol-Myers- Squibb Celgene, and Teva Advisory Board Member. She received congress, and travel/accommodation expense compensations or speaker honoraria from Biogen, Merck, Mylan, Novartis, Sanofi/Genzyme, Teva and Fondazione Italiana Sclerosi Multipla (FISM). Her institutions received research grants from Novartis. The remaining authors declare that the research was conducted in the absence of any commercial or financial relationships that could be construed as a potential conflict of interest.

## Publisher's Note

All claims expressed in this article are solely those of the authors and do not necessarily represent those of their affiliated organizations, or those of the publisher, the editors and the reviewers. Any product that may be evaluated in this article, or claim that may be made by its manufacturer, is not guaranteed or endorsed by the publisher.
