# Supplementary material for: Cannabis-Based Products in a Neurological Setting: A Clinical and Pharmacokinetic Survey
Source: Front Neurol. 2022 Mar 24;13:784748. doi: 10.3389/fneur.2022.784748 (PMC8997238; doi:10.3389/fneur.2022.784748)
Supplement: Supplementary file 1 [file Table_1.DOCX]

Supplementary Material

Table 1. Commercially available cannabis galenical products in Italy.

| Cannabinoid product | Variety | % THC | % CBD |
| --- | --- | --- | --- |
| Bedrocan^®^ | *Cannabis sativa* | 21 | < 1 |
| Bediol^®^ | *Cannabis sativa* | 6.5 | 8 |
| Bedrolite^®^  Bedrobinol^®^  Bedica^®^  FM2 | *Cannabis sativa*  *Cannabis sativa*  *Cannabis sativa*  *Cannabis sativa* | <1  12  14  5-8 | ~ 9  <1  <1  7-12 |

Figure 1. Median plasma concentrations of tetrahydrocannabinol (THC), cannabidiol (CBD)

and matched NRS scores in patients after THC/CBD dosing. Capped bars indicate

25th–75th percentiles.

Table 2. Clinical and therapeutic characteristics of patients who self-assessed their main disease

symptoms by NRS scale pre and post morning CBs dosing.

| Disease | Cannabinoid products | | | |
| --- | --- | --- | --- | --- |
|  | Sativex^®^  (n) | Bediol^®^  (n) | Bedrocan^®^  (n) | Bedrolite^®^  (n) |
| Multiple sclerosis | 13 | 3 | 2 | 1 |
| Neuropathic pain |  | 1 |  |  |
| Spastic paraparesis |  | 1 |  | 2 |
